# Hexokinase 2 dimerization and interaction with voltage‐dependent anion channel promoted resistance to cell apoptosis induced by gemcitabine in pancreatic cancer

**DOI:** 10.1002/cam4.2463

**Published:** 2019-08-19

**Authors:** Kun Fan, Zhiyao Fan, He Cheng, Qiuyi Huang, Chao Yang, Kaizhou Jin, Guopei Luo, Xianjun Yu, Chen Liu

**Affiliations:** ^1^ Department of Pancreatic Surgery Fudan University Shanghai Cancer Center Shanghai P.R. China; ^2^ Department of Oncology, Shanghai Medical College Fudan University Shanghai P.R. China; ^3^ Shanghai Pancreatic Cancer Institute Shanghai P.R. China; ^4^ Pancreatic Cancer Institute Fudan University Shanghai P.R. China

**Keywords:** GEM resistance, HK2 dimer, pancreatic cancer, ROS, VDAC

## Abstract

Gemcitabine (GEM) is the standard chemotherapy drug for pancreatic cancer. Because of widespread drug resistance, the effect is limited. Therefore, it is urgent to reveal the underlying mechanism. Glycolysis is the most remarkable character of tumor aberrant metabolism, which plays vital roles on tumor drug resistance. Hexokinase 2 (HK2), as the key enzyme regulating the first‐step reaction of glycolysis, is overexpressed in many kinds of tumors. The putative role of HK2 resisting GEM therapy was investigated in this study. We found that HK2 was overexpressed in pancreatic cancer and associated with poor prognosis. HK2 knockdown decreased pancreatic cancer cell proliferation, migration viability, and promoted cell apoptosis in vitro. HK2 high expression in pancreatic cancer showed GEM resistance. HK2 knockdown increased the sensitivity of pancreatic cancer cell to GEM, the growth of xenograft tumor with HK2 knockdown was also further decreased with the GEM treatment compared with control in vivo. GEM‐resistant pancreatic cancer showed the increase of HK2 dimer rather than HK2 mRNA or protein. Our study revealed that the ROS derived from GEM promoted HK2 dimerization combining with voltage‐dependent anion channel, which resulted in the resistance to GEM. Meanwhile, our study established a new sight for GEM resistance in pancreatic cancer.

## Graphical abstract

Hexokinase 2 (HK2) overexpression promoted pancreatic cancer growth and gemcitabine (GEM) resistance. ROS mediated the HK2 dimerization induced by GEM.

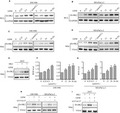

## 1 INTRODUCTION

Pancreatic cancer is a highly aggressive cancer and is characterized by serious drugs resistance and high recurrence^1^, ^2^, ^3^, the 5‐year survival rate is <6%.^1^, ^4^ There are a number of chemotherapeutic drugs employed for pancreatic cancer therapy, which have improved the overall survival of patients.^5^, ^6^, ^7^ Gemcitabine (GEM) is as standard therapeutic for pancreatic cancer for many years.^8^ However, there is common resistance toward GEM for primary or acquired reasons, and many studies have been evaluating GEM resistance in pancreatic cancer.^9^ To elevate GEM‐based treatment, biomarkers were used, including CA19‐9, deoxycytidine kinase, and microRNA; however, these biomarkers have been demonstrated to be insufficient.^6^, ^10^, ^11^ Abnormal expression of several oncoproteins or excessive activation of signaling pathways contribute to GEM resistance^10^, effective strategies have not been provided. Therefore, it is urgent to better understand the underlying molecular mechanism of GEM resistance for pancreatic cancer therapy.

Abnormal metabolism is one of the tumor hallmarks.^12^ To meet the energy and nutrition supply for rapid growth, tumor metabolism processes are reprogrammed, especially glucose metabolism.^12^, ^13^, ^14^ Although the oxygen supply is sufficient, tumor cells prefer to produce ATP via glycolysis, not via mitochondrial oxidation.^12^, ^15^ Abnormal expression or acquired genetic mutations of many metabolic enzymes contribute to tumor glycolysis.^16^, ^17^, ^18^ Hexokinases (HKs), which convert glucose to glucose‐6‐phosphate, are the key enzymes that regulate glycolysis. HKs consist of four isoforms, including HK1, HK2, HK3, and HK4, which are expressed in different tissues.^19^ HKs sustain cellular glucose level by regulating the cellular entry and utilization of glucose. Therefore, HKs have an effect on the cellular glucose flux and energy supply.^20^, ^21^ HK2 is abundantly expressed in embryonic tissues but is limited in adult tissues. A high level of HK2 expression is found in many types of tumors.^22^ HK2 regulates tumor cellular glucose metabolism to support cell proliferation, migration, and apoptosis resistance, which is required for tumor initiation and development.^19^

VDAC is the voltage‐dependent anion channel and is located at the outer membrane of mitochondria. VDAC controls the entry and exit of ions and metabolites between the cytosol and mitochondria through the “open” and “closed” states.^23^ When the difference in membrane potential is within +20 to −20 mV, VDAC is in the “open” state.^24^, ^25^, ^26^ When the difference in membrane potential is lower than −20 mV or higher than +20 mV, VDAC switches to the “closed” state.^24^, ^25^, ^26^ Three isoforms of VDAC have been identified, including VDAC1, VDAC2, and VDAC3.^27^ Only VDAC1 is widely expressed, and it represents 5% of total mitochondrial proteins.^27^ Considering that VDAC maintains the mitochondrial membrane potential (MMP) and prevents the release of cytochrome c, it plays an important role in antiapoptosis.^28^ It has been reported that the overexpression of HK2 and its interaction with VDAC are required for resisting cell apoptosis.

The study described in this report established the critical role of HK2 overexpression in resistance to GEM‐induced cell apoptosis and elucidated the underlying mechanism in pancreatic cancer, which demonstrated that HK2 deletion increases sensitivity to GEM therapy.

## 2 MATERIALS AND METHODS

### 2.1 Cell culture

Human pancreatic cancer cell lines BxPC‐3, PANC‐1, Capan‐1, MIAPaCa‐2, and SW1990 were purchased from the American Type Culture Collection. All cells were cultured with DMEM or 1640 medium with 100 U/mL penicillin, 100 μg/mL streptomycin, 10% fetal bovine serum at 37°C under 5% CO_2_.

### 2.2 Pancreatic cancer tissue samples and tissue microarray

The pancreatic cancer tissue microarray (TMA) and samples were obtained from Pancreas Surgery of Fudan University Shanghai Cancer Center from 2010 to 2012. Pancreatic cancer samples were analyzed. Prior patient consent and approval from the Institutional Research Ethics Committee were obtained.

### 2.3 Antibodies and reagents

HK2 and GAPDH antibody (Proteintech, Cat.#22029‐1‐AP), VDAC antibody (Abcam,Cat.#ab14734) were purchased from Abcam (Abcam), GEM was bought from MedChemExpress (MedChemExpress, Cat.# HY‐17026).

### 2.4 Western blot

Cells were prepared, treated, and collected for protein extraction. Briefly, cells in the dish were washed once with phosphate buffer saline (PBS) and lysed at 4°C with radio‐immunoprecipitation assay (RIPA) lysis. The mixture was centrifuged and the supernatants were collected as protein samples. The protein concentration was measured by the bicinchoninic acid (BCA) protein concentration assay kit (KANGWEI, Shanghai, China, Cat.#CW0014S). Protein samples with same mass were prepared, separated via SDS‐PAGE, and transferred onto Polyvinylidene Fluoride membranes. Then the membrane was blocked with 5% nonfat milk and incubated with the specific primary antibody at 4°C. Next, the membranes were washed with phosphate buffered saline tween‐20 (PBST) and incubated with the corresponding horseradish peroxidase (HRP)‐conjugated secondary antibody. The membranes were washed with PBST. The target protein was finally visualized using an enhanced chemiluminescence system.

### 2.5 Real‐time PCR

Cells were treated and total RNA was isolated by Trizol (Invitrogen, NY, Cat. #15596026) according to the manufacturer's instructions. Next, reverse transcription was performed using the Takara reverse transcription kit (Takara, Japan, Cat.#RR036A). Real‐time PCR was performed using SYBR Green (Takara, Japan, Cat.#RR420A) reagent according to the manufacturer's instructions. The reaction system consisted of cDNA 4.5 μL (100 ng/μL), 2× SYBR mix 5 μL, forward primer and reverse primer 0.2 μL each, and ROX 0.1 μL; the total volume was 10 μL. Finally, the HK2 mRNA expression level was calculated. Real‐time PCR detection was performed in at least three independent experiments. The HK2 forward primer sequence: TCAATATTAGAGTCTCAACCCCCA and reverse primer sequence GAAGGCGCTTGTGGAGAAGG.

### 2.6 Immunohistochemical staining

Immunohistochemistry (IHC) was performed as previously described.^24^ The images were captured at 400× magnification. The percentage of the positive cells was divided into five grades: <10% (0), 10%‐25% (1), 25%‐50% (2), 50%‐75% (3), and >75% (4). The intensity of staining was divided into four grades: no staining (0), light brown (1), brown (2), and dark brown (3). The IHC staining score was calculated according to the percentage and the intensity divided into −, +, ++, and +++ as negative, weakly positive, positive, and strongly positive, respectively.

### 2.7 Stable cell strain establishment

The target sequence of shHK was given below: shHK2f: CCGGTCCAAAGACATCTCAGACATTGTTCAAGAGACAATGTCTGAGATGTCTTTGGTTTTTG, shHK2r:AATTCAAAAACCAAAGACATCTCAGACATTGTCTCTTGAACAATGTCTGAGATGTCTTTGGA, shNC‐f:CCGGTTTCTCCGAACGTGTCACGTTTCAAGAGAACGTGACACGTTCGGAGAATTTTTG, shNC‐r:AATTCAAAAATTCTCCGAACGTGTCACGTTCTCTTGAAACGTGACACGTTCGGAGAAA. The sequences including 62 bp were synthesized and constructed into pLKO.1‐puro vector at AgeI and EcoRI sides. The constructed plasmids were sequenced, and the sequence was confirmed to be correct. Lentiviruses were generated by cotransfection of pGAG, pVSVG, and pLKO.1‐shHK2 according to the ratio 3:3:5 into 293T cells with NanoEnter (New Cell& Molecular Biotech Co., Ltd). To collect lentivirus particles, the supernatant was centrifuged at 300 *g* to remove cell debris and filtered through a 0.45‐μm filter (Merck Millipore). MIAPaCa‐2 cells were transfected with lentivirus particles expressing shHK2 or scrambled nontarget shNC. For screening the stable cells, puromycin (2 μg/mL) (MedChemExpress, Cat.#HY‐B1743) was added into cells after lentivirus infection. For control, the nontarget shNC was transfected, and cells were selected with puromycin. The HK2 knockdown efficiency was verified by western blot analysis.

### 2.8 Cell proliferation viability assay

Cells were digested and the cell concentration was adjusted at 10^6^ cells/mL. Next, the cells were plated into a 96‐well plate with 2000 cells per well for cell viability assay and a 6‐well plate with 1000 cells per well for clone formation assay. Cell viability assay was completed by CCK8 and the proliferation curve was calculated. Cell clones were counted by crystal violet staining.

### 2.9 Transwell assay

Cells were digested and suspended in serum‐free medium at a concentration of 10^6^ cells/mL. The transwell chamber was placed into the wells of a 24‐well plate, and medium containing 10% serum was added into the bottom of the chamber. Cell suspension with approximately 10^5^ cells was added into the chamber and cultured for approximately 24 hours. Finally, the chamber was stained with crystal violet, and the number of transferred cells was calculated.

### 2.10 Flow cytometry analysis of apoptosis

Cells for apoptosis detection were plated into a six‐well plate. Following treatment completion, the cells were digested and washed once with PBS. The cell staining was completed according to the manufacturer's instructions. Briefly, the sample was resuspended with 195 μL of apoptosis staining buffer, 5 μL of Annexin V‐fluorescein isothiocyanate was added and mixed, and 10 μL of PI was added and mixed. The mixture was incubated away from light at room temperature for 20 minutes. Finally, the cell suspension was assayed via flow cytometry.

### 2.11 Xenograft tumor growth assay in vivo

Animal experiments were conducted with the approval of the animal ethics committee of Fudan University. Pancreatic cancer cells were digested and suspended with cold PBS at a density of 10^7^ cells/mL. Approximately, 100 μL of cell suspension with 10^6^ cells was injected subcutaneously into 4‐ to 5‐week‐old female or male Balb/C nude mice in the right and left abdomen. The xenograft tumor growth was monitored by the measurement of long diameter and short diameter, and tumor volume was calculated as described, *V* (mm^3^) = width^2^ (mm^2^) × length (mm)/2. Finally, the tumors were harvested and analyzed.

### 2.12 HK2 dimer assay

Glutaraldehyde cross‐linking assay was employed to analyze HK2 dimerization (Figure S1).

Glutaraldehyde solution was firstly prepared. About 10‐μL glutaraldehyde solution 50 wt.% (Sigma Aldrich #340855) was added to 990‐μL double‐distilled H_2_O as stock solution. Cells from 6‐cm dishes with about 90% cell density were scraped and lysed in 400‐μL 0.1% NP‐40 lysis buffer with phenylmethanesulfonyl fluoride (PMSF) at 4°C for 30 minutes, and centrifuged at 12000 *g* for 20 minutes at 4°C Then 200‐μL lysate of each sample was drawn into two tubes. One tube was as control. The other one was added 10‐μL 0.5% glutaraldehyde stock solution to the lysate and incubated on ice for 5 minutes. About 10‐μL 1 mol/L glycine was added for 15 minutes at room temperature for quenching glutaraldehyde. Each tube was added 50‐μL 5× loading buffer and boiled at 100°C for 10 minutes. Finally, the sample was detected by western blot.

### 2.13 Statistics

The significant differences between the mRNA and protein level were assessed via Student's *t* test. The statistical results were presented as the mean ± SD from experiments conducted in at least triplicates. Differences were considered to be significant when *P* < .05 for all statistical analyses.

## 3 RESULTS

### 3.1 HK2 was overexpressed in pancreatic ductal adenocarcinoma

**Figure 1:**
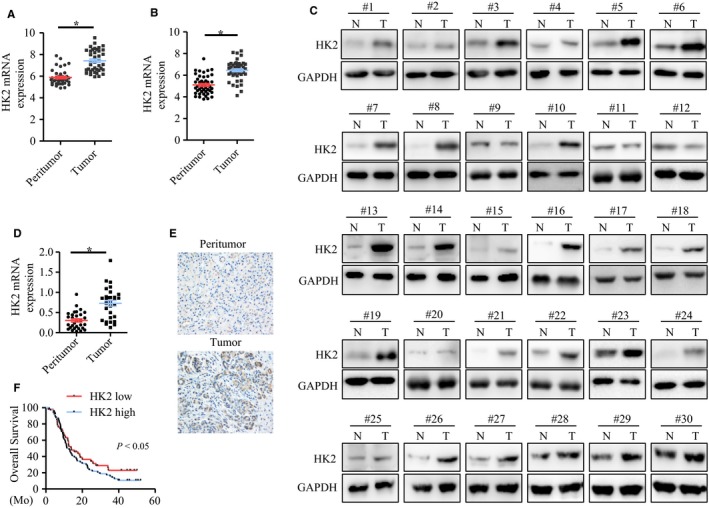
HK2 was overexpressed in pancreatic cancer. A,B, HK2 expression in pancreatic cancer and peritumor tissues, from GEO15471 and GEO287735 databases. C, Western blot analysis of HK2 expression in 30 pairs of pancreatic cancer with corresponding peritumor tissues. D, The statistical analysis of HK2 expression in pancreatic cancer and peritumor tissues. E, immunohistochemistry analysis of HK2 expression in pancreatic cancer and peritumor tissues. F, Kaplan‐Meier analysis of overall survival of pancreatic cancer patients with high and low HK2 expression. Abbreviation: HK2, hexokinase 2

To establish the expression of HK2 in pancreatic cancer, we analyzed the GEO database. From GSE15471 with 39 pairs of pancreatic tumor and peritumor tissues, we found that HK2 expression was higher in pancreatic tumors compared with the corresponding peritumor tissue (*P* < .05) (Figure 1A). From GSE287735, HK2 expression was also higher in pancreatic tumors than that in peritumor tissue (*P* < .05) (Figure 1B). Next, we detected 30 pairs of pancreatic tumor samples with peritumor tissues via western blot. The result showed that HK2 expression was higher in tumor samples compared with peritumor tissues (Figure 1C,D). To further explore HK2 expression in pancreatic cancer samples, we analyzed the pancreatic tumor TMA with 108 samples and found that HK2 was commonly expressed in the tumors. About 70% tumors showed HK2‐positive staining, and rarely, approximately 20% peritumor tissues showed weak positive staining (Figure 1E). To determine the role of HK2, the overall survival was analyzed. Patients with HK2 high expression showed poorer prognosis than those with HK2 low expression (Figure 1F). The results suggested that HK2 possibly played important roles in pancreatic cancer.

### 3.2 HK2 knockdown decreased pancreatic cancer cell proliferation and migration, but increased cell apoptosis

**Figure 2:**
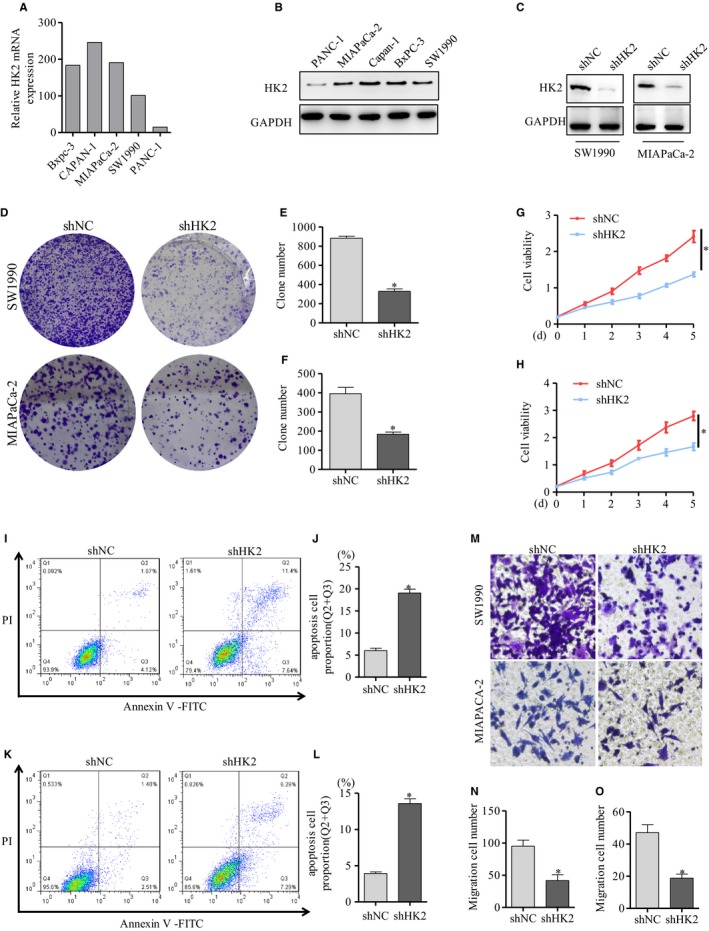
HK2 knockdown decreased cell proliferation and migration, but increased cell apoptosis. A, HK2 expression of pancreatic cancer cells from Betastasis database. B, Western blot analysis of HK2 expression in pancreatic cancer cell lines. C, Western blot detection of HK2 knockdown efficiency mediated by shRNA in SW1990 and MIACaPa‐2 cells. D, The clone formation assay of cell proliferation in SW1990 and MIACaPa‐2 cells with HK2 knockdown. E,F, The statistical analysis of cell clone formation in SW1990 and MIACaPa‐2 cells with HK2 knockdown. G,H, Cell viability was detected by CCK8 in SW1990 and MIACaPa‐2 cells with HK2 knockdown. I, Cell apoptosis was detected via flow cytometry. J, Cell apoptosis result was calculated in SW1990 cells with HK2 knockdown. K, Cell apoptosis was detected and L, result was calculated in MIAPaCa‐2 cells with HK2 knockdown. M, Cell migration was detected by transwell in SW1990 and MIAPaCa‐2 cells with HK2 knockdown. N,O, The statistical analysis of cell transwell assay in SW1990 and MIAPaCa‐2 cells with HK2 knockdown. The results represented three independent sets of experiments. Abbreviation: HK2, hexokinase 2

To confirm the role of HK2 in pancreatic cancer cells, we analyzed HK2 expression in five pancreatic cancer cell lines via western blot analysis. HK2 was commonly expressed (Figure 2B). A high expression of HK2 was observed in MIAPaCa‐2, Capan‐1, Bxpc‐3, and SW1990 cells, while HK2 low expression was observed in PANC‐1 cells (Figure 2B), which was consistent with the HK2‐expression data from the database (Figure 2A). To verify the function of HK2 in pancreatic cancer, knockdown of HK2 was completed in MIAPaCa‐2 and SW1990 cells. Western blot results showed that the knockdown efficiency was approximately 90% and 85% in the shHK2 cell strains of SW1990 and MIAPaCa‐2, respectively (Figure 2C). Clone formation was performed for the analysis of cell proliferation (Figure 2D). The results showed that cell proliferation was decreased by approximately 63% (Figure 2E) and 54% (Figure 2F) in the shHK2 cell strains of SW1990 and MIAPaCa‐2 cells, respectively. Cell viability was determined via CCK8 assay. The results showed that cell viability was decreased by approximately 43% and 40% in the shHK2 cell strains of SW1990 and MIAPaCa‐2 cells, respectively (Figure 2G,H). Flow cytometry was conducted to detect cell apoptosis in SW1990 and MIAPaCa‐2 cells (Figure 2I,K). The statistical results showed that the proportion of apoptosis was 5.99% in shNC and 19.04% in shHK2 of SW1990 cells (Figure 2J), 3.91% in shNC and 12.58% in shHK2 of MIAPaCa‐2 cells (Figure 2L). Cell apoptosis was increased after HK2 knockdown. We further analyzed the effect of HK2 on cell migration (Figure 2M). After HK2 knockdown, cell migration was decreased by approximately 59% and 61% in the shHK2 cell strains of SW1990 and MIAPaCa‐2 cells (Figure 2N,O). These results showed that HK2 knockdown decreased cell proliferation, migration, and promoted cell apoptosis in pancreatic cancer.

### 3.3 HK2 knockdown enhanced pancreatic cancer cell sensitivity to GEM in vitro

**Figure 3:**
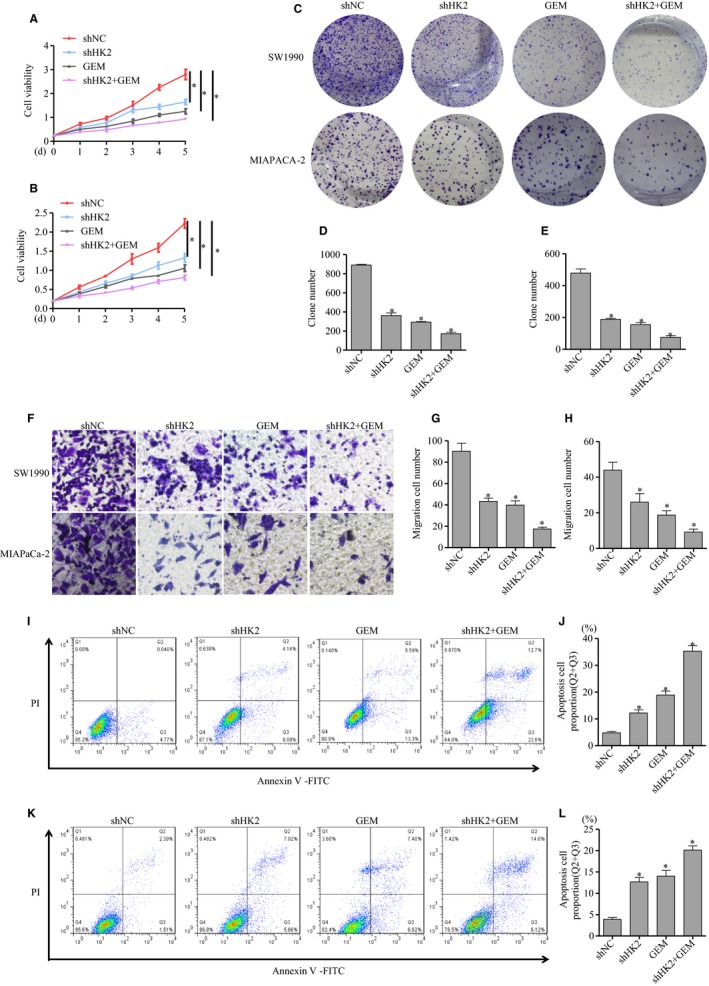
HK2 knockdown increased the sensitivity to GEM. The stable cell strains with HK2 knockdown were established in SW1990 and MIAPaCa‐2 cells, then treated by GEM. A,B, The cell viability was assayed via CCK8 in SW1990 and MIAPaCa‐2 cells. C, The clone formation assay of cell proliferation capacity in SW1990 and MIAPaCa‐2 cells. D,E, The statistical analysis of clone formation in SW1990 and MIAPaCa‐2 cells. F, Transwell assay of cell migration in SW1990 and MIAPaCa‐2 cells. G,H, The statistical analysis of cell transwell assay in SW1990 and MIAPaCa‐2 cells. I,K, Cell apoptosis analysis via flow cytometry J,L, results were calculated in SW1990 and MIAPaCa‐2 cells. The results represented three independent sets of experiments. Abbreviations: GEM, gemcitabine; HK2, hexokinase 2

Given that HK2 promoted cell proliferation and resistance to apoptosis in pancreatic cancer cells, we analyzed the response to GEM therapy when HK2 was knocked down. Cells were divided into four groups with different treatments: shNC, shHK2, GEM, and shHK2 with GEM. Compared with shNC group, the cell viability was decreased by approximately 40% in the shHK2 group, 52% in GEM treatment group, and 63% for the shHK2 with GEM treatment in the SW1990 cells (Figure 3A). Similarly, the cell viability was decreased by approximately 41% in the shHK2 group, 55% in GEM treatment group, and 66% for shHK2 with GEM treatment in MIAPaCa‐2 cells (Figure 3B). The cell viability was more significantly decreased in the shHK2 group with GEM treatment compared with that in other groups. Consistently, the clone formation capacity showed 60% reduction in the shHK2 group, 67% reduction in GEM treatment group, and 80% reduction in shHK2 with GEM treatment compared with shNC group in SW1990 cells (Figure 3C,D). Similarly, in the MIAPaCa‐2 cells, the clone formation viability showed approximately 61% reduction in the shHK2, 68% reduction in GEM treatment, and 85% reduction in the shHK2 with GEM treatment compared with shNC (Figure 3C,E). The cell migration viability was analyzed via transwell. Results showed that cell migration viability was approximately 53% reduction in the shHK2 group, 56% reduction in the GEM treatment group, and 81% reduction in shHK2 with GEM treatment compared with shNC in SW1990 cells (Figure 3F,G). In MIAPaCa‐2 cells, the cell migration viability was approximately 60% reduction in the shHK2 group, 76% reduction in the GEM treatment group, and 80% reduction in shHK2 with GEM treatment compared with shNC (Figure 3F,H). The cell migration viability was decreased more significantly in shHK2 with GEM treatment group than that in other groups. To further assess the role of HK2 in cell apoptosis induced by GEM, flow cytometry was accomplished in SW1990 and MIAPaCa‐2 cells (Figure 3I,K). The statistical results showed the proportion of apoptosis was approximately 4.81% in shNC group, 12.22% in shHK2 group, 18.89% in shNC with GEM treatment, and 35.3% in shHK2 with GEM treatment in SW1990 cells (Figure 3J). Similarly, in the MIAPaCa‐2 cells, the proportion of apoptosis was approximately 3.9% in the shNC group, 12.68% in the shHK2 group, 14.02% in shNC with GEM treatment, and 20.12% in shHK2 with GEM treatment (Figure 3L).They suggested that HK2 knockdown promoted the sensitivity of pancreatic cells to GEM.

### 3.4 GEM did not affect HK2 expression at transcriptional and translational levels

**Figure 4:**
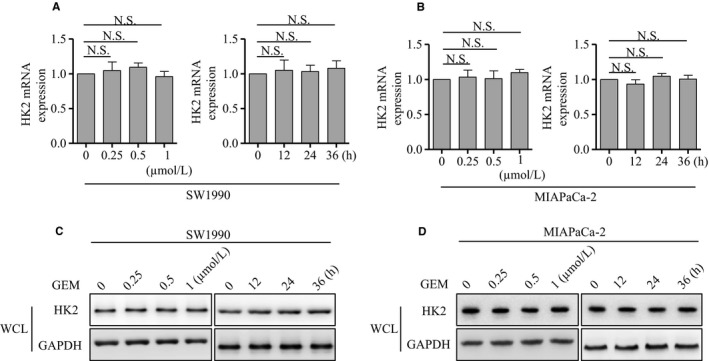
GEM did not affect HK2 mRNA and protein level. A, Real‐time PCR analysis of HK2 mRNA expression after GEM treatment at the indicated concentration and time in SW1990 cells. B, Real‐time PCR analysis of HK2 mRNA expression after GEM treatment at the indicated concentration and time in MIAPaCa‐2 cells. C, Western blot analysis of HK2 monomer levels after GEM treatment at the indicated concentration and time in the whole cell lysis of SW1990 cells. D, Western blot analysis of HK2 monomer level after GEM treatment at the indicated concentration and time in the whole cell lysis of MIAPaCa‐2 cells. The results represented three independent sets of experiments. Abbreviations: GEM, gemcitabine; HK2, hexokinase 2

To explore the underlying mechanism, we detected the mRNA and protein expression of HK2 after GEM treatment. The mRNA was not significantly changed after GEM treatment in SW1990 and MIAPaCa‐2 cells (Figure 4A,B). Similarly, the protein expression was also not significantly changed in SW1990 and MIAPaCa‐2 cells (Figure 4C,D). The results suggested that GEM did not affect HK2 expression at the transcriptional and translational levels.

### 3.5 GEM induced the increase of HK2 dimerization via ROS

**Figure 5:**
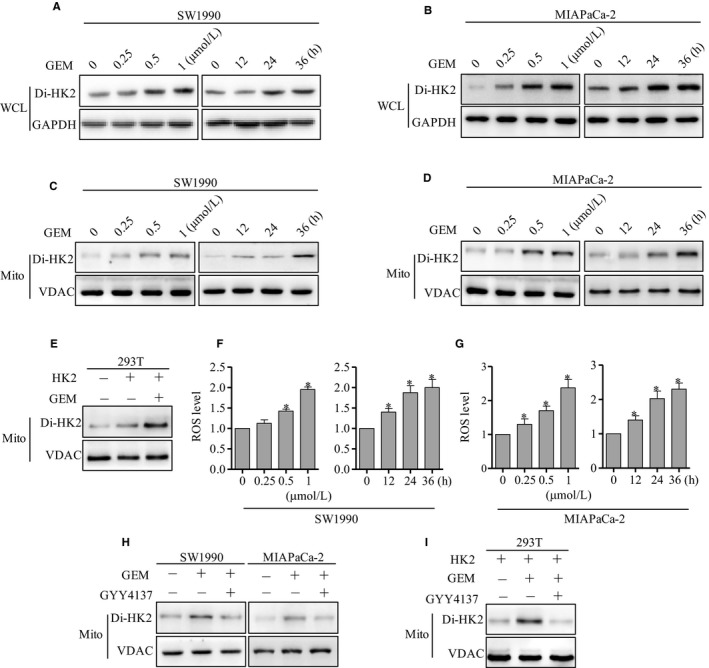
GEM increased the HK2 dimer level via ROS. A, Western blot analysis of HK2 dimer level in total cell lysis buffer after GEM treatment at the indicated concentration and time in SW1990 cells. B, Western blot analysis of HK2 dimer level in total cell lysis buffer after GEM treatment at the indicated concentration and time in MIAPaCa‐2 cells. C, Western blot analysis of HK2 dimer level in mitochondrion after GEM treatment at the indicated concentration and time in SW1990 cells. D, Western blot analysis of HK2 dimer level in mitochondrion after GEM treatment at the indicated concentration and time in MIAPaCa‐2 cells. E, HK2 was transfected into 293T cells, then treated by GEM, the HK2 dimer was detected by western blot. F, ROS level was detected after GEM treatment at the indicated concentration and time in SW1990 cells. G, ROS level was detected after GEM treatment at the indicated concentration and time in MIAPaCa‐2 cells. H, SW1990 and MIAPaCa‐2 cells were treated by GEM and GYY4137, the HK2 dimer was detected by western blot. I, HK2 was transfected into 293T cells, then treated by GYY4137 before GEM, the HK2 dimer was detected by western blot. The results represented three independent sets of experiments. Abbreviations: GEM, gemcitabine; HK2, hexokinase 2; VDAC, voltage‐dependent anion channel

HK2 exists the monomer and homodimer formation. Next, we analyzed whether GEM had an effect on HK2 dimerization. The western blot results showed that HK2 dimer was significantly increased after GEM treatment in SW1990 and MIAPaCa‐2 cells at concentration‐ or time‐dependent way (Figure 5A,B). HK2 existed the mitochondrion location.^18^, ^19^ Next, mitochondrion was isolated and HK2 dimer was detected. The results showed that HK2 dimer was also increased after GEM treatment in SW1990 and MIAPaCa‐2 cells at concentration‐ or time‐dependent way in mitochondrion (Figure 5C,D). To further determine the role of GEM on HK2 dimerization, 293T cells were transfected with HK2 plasmid, then treated with GEM, the HK2 dimer was significantly increased (Figure 5E). Reactive oxygen species (ROS) was the key downstream effector of GEM.^29^, ^30^ Next, we detected the ROS level and found that it was significantly increased after GEM treatment in SW1990 (Figure 5F) and MIAPaCa‐2 cells (Figure 5G). It was unknown whether ROS played important roles on HK2 dimerization. To determine the role of ROS, GYY4137,^31^ a reductant reducing the cellular ROS, was employed. When cells were pretreated with GYY4137, then treated by GEM, the HK2 dimer was reduced compared with only GEM treatment in SW1990 cells (Figure 5H). Consistently, when MIAPaCa‐2 cells were pretreated with GYY4137, the HK2 dimer was also reduced compared with GEM group (Figure 5H). 293T cells were transfected with HK2 plasmid, then pretreated by GYY4137 before GEM, the increase of HK2 dimer by GEM was also significantly inhibited (Figure 5I). The results indicated that ROS possibly mediated the HK2 dimerization by GEM.

### 3.6 HK2 dimerization promoted interaction with VDAC

**Figure 6:**
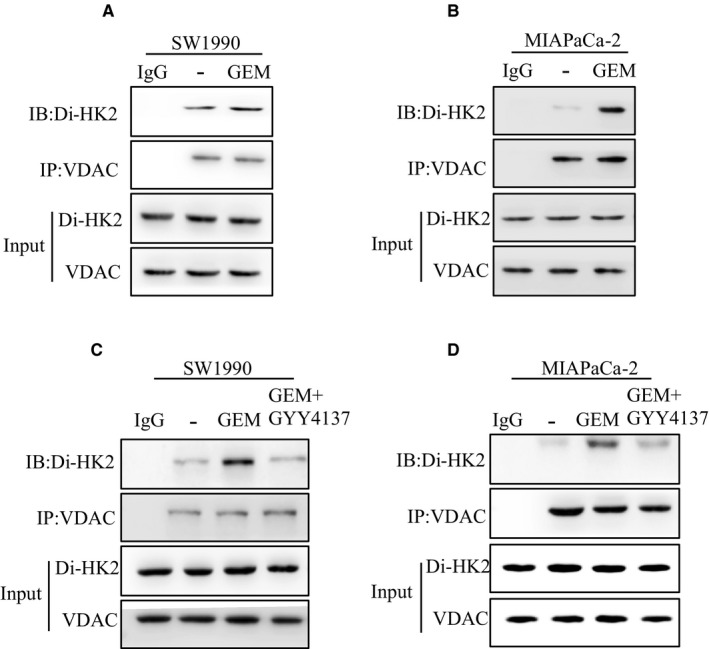
The increase of HK2 dimer by GEM promoted its combining with VDAC. A, SW1990 cells were treated by GEM, mitochondrion was isolated and lysed. The interaction of HK2 dimer and VDAC was analyzed via co‐immunoprecipitation. B, MIAPaCa‐2 cells were treated by GEM, mitochondrion was isolated and lysed. The interaction of HK2 dimer and VDAC was analyzed via co‐immunoprecipitation. C, SW1990 cells were treated by GEM and GYY4137, mitochondrion was isolated and lysed. The interaction of HK2 dimer and VDAC was analyzed via co‐immunoprecipitation. D, MIAPaCa‐2 cells were treated by GEM and GYY4137, mitochondrion was isolated and lysed. The interaction of HK2 dimer and VDAC was analyzed via co‐immunoprecipitation. Abbreviations: GEM, gemcitabine; HK2, hexokinase 2; VDAC, voltage‐dependent anion channel

It has been reported that HK2 combining with VDAC inhibited cell apoptosis.^23^ To explore the role of HK2 dimerization, we detected the interaction of HK2 with VDAC via co‐immunoprecipitation. From the results, GEM treatment increased the HK2 dimer combining with VDAC in SW1990 cells (Figure 6A). Moreover, GEM treatment increased the HK2 dimer combining with VDAC in MIAPaCa‐2 cells (Figure 6B). We further analyzed the role of ROS on HK2 combining with VDAC. When cells were pretreated by GYY4137 before GEM, the HK2 dimer combining with VDAC was decreased in SW1990 cells (Figure 6C). Moreover, GYY4137 pretreatment before GEM also decreased the HK2 dimer combining with VDAC in MIAPaCa‐2 cells (Figure 6D). These suggested that the increase of HK2 dimer by GEM promoted to combine with VDAC.

### 3.7 HK2 knockdown enhanced pancreatic cancer sensitivity to GEM therapy in vivo

**Figure 7:**
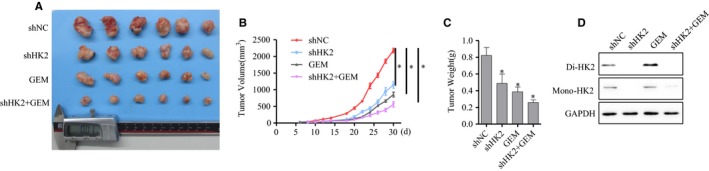
Xenograft tumor growth was more significantly inhibited by GEM after HK2 knockdown. A, The samples of xenograft tumor were injected subcutaneously and grew for 30 d with GEM treatment. B, The growth curve of xenograft tumor was calculated from the initiation of xenograft tumor growth to harvest. C, The weight of xenograft tumor was calculated. D, Western blot analysis of HK2 dimer in xenograft tumor. Abbreviations: GEM, gemcitabine; HK2, hexokinase 2

To further explore the role of HK2 in GEM treatment in vivo, a mouse subcutaneous xenograft tumor model was completed. The mice were divided into four groups including shNC, shHK2, GEM treatment, and shHK2 with GEM treatment. Nude mice were injected subcutaneously with10^6^ cells. GEM was administered via tail intravenous injection every other day in the groups of GEM treatment and shHK2 with GEM treatment. The shNC and shHK2 groups were injected saline as control. The xenograft tumors were monitored and measured every other day for evaluating the xenograft tumor growth. After 30 days, the xenograft tumors were harvested for further analysis (Figure 7A). The tumor growth curve (Figure 7B) and weight (Figure 7C) were calculated. From the tumor growth curve, we found that GEM treatment decreased the xenograft growth. The xenograft tumor with HK2 knockdown was more sensitive to GEM treatment, and the growth and weight of shHK2 with GEM treatment tumor were decreased more significantly compared with GEM group. Western blot demonstrated that HK2 expression was also decreased in the shHK2 group xenograft tumor and GEM treatment also increased HK2 dimer in vivo (Figure 7D). These results suggested that HK2 knockdown increased the sensitivity of pancreatic cancer cells to GEM therapy.

## 4 DISCUSSION

Metabolism reprogramming has been identified as a hallmark of cancer, this effect results in a high flux of glucose utilization in tumors to support rapid tumor growth.^12^ Therefore, the abnormal or selective cancer‐associated expression of isoforms of glycolytic enzymes enables the attainment of the required amount of energy.^20^, ^21^ HK2 catalyzes the first‐step reaction of glycolysis and controls the glucose flux into glycolysis.^19^ It has been reported that HK2 is specifically expressed in the tumor and plays critical roles in tumor initiation and development.^19^ HK2 promotes tumor growth, migration, and angiogenesis via mediating aerobic glycolysis in a xenograft tumor model.^19^, ^32^ Other studies also show that HK2 is required for tumor initiation and maintenance in an HK2‐conditional knockout mouse model.^33^, ^34^ However, it is unknown whether these results could be extrapolated to pancreatic cancer. GEO database showed that HK2 was overexpressed in pancreatic cancer. Analysis of pancreatic cancer clinical samples via western blot and real‐time PCR further confirmed the results. HK2 located on the mitochondria is coupled with oxidative phosphorylation, which facilities ATP acquirement of HK2 and elevates glycolysis to achieve rapid tumor growth.^35^ Our cell experiment suggested that HK2 promoted pancreatic cancer cell proliferation and migration in vitro. The tumor xenograft growth was inhibited after HK2 knockdown in vivo. The survival analysis showed that patients of HK2 high expression had poorer prognosis than those of HK2 low expression.

Overexpression of HK2 protects cancer cells against apoptosis induced by oxidants or other stimuli.^36^ One hypothesis explains that interaction of HK2 with VDAC is responsible for antiapoptosis.^23^, ^26^, ^36^ Given the importance of VDAC in metabolism and cell death, many studies on VDAC have become the focus. Because it controls the release of cytochrome c, VDAC is a critical regulator of cell apoptosis.^23^, ^25^ It has been reported that HK2 promoted the phosphorylation of VDAC and further interacted with VDAC through the protein kinase C (PKC) pathway, which maintains the MMP against apoptosis.^23^, ^25^ In our research, HK2 knockdown by shRNA increased apoptosis in pancreatic cancer cells. Because of the antiapoptosis activity of HK2, the drug resistance of tumors was attributed to HK2 overexpression. However, it was unclear whether HK2 was responsible for GEM resistance in pancreatic cancer.

It has been reported that GEM resistance is common in primary or acquired pancreatic cancers.^9^, ^37^ Considering that GEM is a basic therapy, several studies have explored the underlying mechanism of GEM resistance.^9^, ^37^ Our results suggested that HK2 knockdown increased cell apoptosis compared with only GEM treatment, which made pancreatic cancer cells more sensitive to GEM. To further analyze the mechanism of HK2 resistance to GEM, we detected the HK2 mRNA and protein level after GEM treatment. The HK2 mRNA and protein level were not obviously changed after GEM treatment, which suggested that GEM did not affect the HK2 transcription or translation. HK2 has the monomer and dimer formation and plays important roles. Our exploration found that GEM could increase HK2 dimer in pancreatic cancer cells. Moreover, overexpression of HK2 in 293T cells also upregulated the HK2 dimer level. This indicated that HK2 dimerization induced by GEM indirectly promoted the GEM resistance. As the key downstream effector of GEM, ROS plays key role on affecting cell apoptosis.^29^, ^30^ We speculated that ROS mediated the GEM‐induced HK2 dimerization. Therefore, we employed GYY4137 to pretreat pancreatic cancer cells before GEM, results showed that HK2 dimer induced by GEM was inhibited in SW1990 and MIAPaCa‐2 cells. HK2 overexpression in 293T cells was pretreated with GYY4137 before GEM, the HK2 dimer was consistently decreased. The results indicated that ROS produced by GEM promoted HK2 dimerization.

Previous studies have demonstrated that the antiapoptosis role of HK2 was responsible for the interaction with VDAC. They have reported that AKT, as an important regulator, promotes HK2 translocation from the cytoplasm to mitochondria; therefore, targeting AKT inhibition was believed to be feasible. GEM treatment promoted HK2 dimerization rather than HK2 mRNA and protein expression. It has been reported that HK2 interacts with VDAC in polymer form.^23^, ^25^ The further exploration determined that the increase of HK2 dimer promoted its interaction with VDAC. GYY4137 pretreatment could inhibit the interaction with VDAC by reducing ROS level. Therefore, the results suggested that ROS produced by GEM promoted HK2 dimerization and its combining with VDAC, which inhibited cell apoptosis induced by GEM.

It is known that embryonic and cancer cells preferentially use aerobic glycolysis to support proliferation.^18^, ^19^ HK2 is mainly expressed in embryonic tissues but less expressed in adult tissues.^19^ However, HK2, not other HKs, is selectively overexpressed in many tumors, and it plays a critical role in these tumors.^19^ The overexpression of HK2 in pancreatic cancer was demonstrated to promote pancreatic cancer growth and GEM resistance, thereby providing a new strategy for enhancing the sensitivity to GEM via targeting HK2.

## CONFLICT OF INTEREST

The authors declare no conflict of interest.
