# Supplementary figures and images for: Hexokinase 2 dimerization and interaction with voltage‐dependent anion channel promoted resistance to cell apoptosis induced by gemcitabine in pancreatic cancer
Source: Cancer Med. 2019 Aug 19;8(13):5903–15. doi: 10.1002/cam4.2463 (PMC6792491; doi:10.1002/cam4.2463)

+Glutaraldehyde

-Glutaraldehyde

SW1990

CAPAN1

Bxpc3

SW1990

CAPAN1

Bxpc3

250

130

100

70

55

35

25

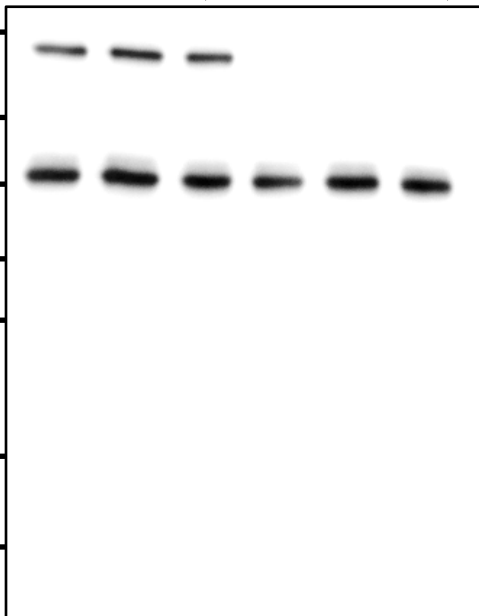

Supplement: Supplementary file 1 [file CAM4-8-5903-s001.pdf]
